# Duffy Negative Antigen Is No Longer a Barrier to *Plasmodium vivax* – Molecular Evidences from the African West Coast (Angola and Equatorial Guinea)

**DOI:** 10.1371/journal.pntd.0001192

**Published:** 2011-06-21

**Authors:** Cristina Mendes, Fernanda Dias, Joana Figueiredo, Vicenta Gonzalez Mora, Jorge Cano, Bruno de Sousa, Virgílio E. do Rosário, Agustin Benito, Pedro Berzosa, Ana Paula Arez

**Affiliations:** 1 Centro de Malária e Outras Doenças Tropicais, Unidade de Parasitologia, Instituto de Higiene e Medicina Tropical, Universidade Nova de Lisboa, Lisbon, Portugal; 2 Departamento de Medicina Interna, Faculdade de Medicina, Universidade Agostinho Neto, Luanda, Angola; 3 Centro Nacional de Medicina Tropical, Instituto de Salud Carlos III, Madrid, Spain; 4 Centro de Malária e Outras Doenças Tropicais, Unidade de Saúde Internacional, Instituto de Higiene e Medicina Tropical, Universidade Nova de Lisboa, Lisbon, Portugal; Emory University, United States of America

## Abstract

**Background:**

*Plasmodium vivax* shows a small prevalence in West and Central Africa due to the high prevalence of Duffy negative people. However, Duffy negative individuals infected with *P. vivax* have been reported in areas of high prevalence of Duffy positive people who may serve as supply of *P. vivax* strains able to invade Duffy negative erythrocytes. We investigated the presence of *P. vivax* in two West African countries, using blood samples and mosquitoes collected during two on-going studies.

**Methodology/Findings:**

Blood samples from a total of 995 individuals were collected in seven villages in Angola and Equatorial Guinea, and 820 *Anopheles* mosquitoes were collected in Equatorial Guinea. Identification of the *Plasmodium* species was achieved by nested PCR amplification of the small-subunit rRNA genes; *P. vivax* was further characterized by *csp* gene analysis. Positive *P. vivax*-human isolates were genotyped for the Duffy blood group through the analysis of the *DARC* gene. Fifteen Duffy-negative individuals, 8 from Equatorial Guinea (out of 97) and 7 from Angola (out of 898), were infected with two different strains of *P. vivax* (VK210 and VK247).

**Conclusions:**

In this study we demonstrated that *P. vivax* infections were found both in humans and mosquitoes, which means that active transmission is occurring. Given the high prevalence of infection in mosquitoes, we may speculate that this hypnozoite-forming species at liver may not be detected by the peripheral blood samples analysis. Also, this is the first report of Duffy negative individuals infected with two different strains of *P. vivax* (VK247 and classic strains) in Angola and Equatorial Guinea. This finding reinforces the idea that this parasite is able to use receptors other than Duffy to invade erythrocytes, which may have an enormous impact in *P. vivax* current distribution.

## Author Summary

Recent reports of *Plasmodium vivax* infections, the most widely distributed species of human malaria, show that this parasite is evolving and adapting, becoming not only more aggressive but also more frequent in countries where it was not present in the past, becoming, therefore, a major source of concern. Thus, it is extremely important to perform new studies of its distribution in West and Central Africa, where there are few reports of its presence, due to the high prevalence of Duffy-negative individuals. The aim of this study was to investigate the presence of *P. vivax* in Angola and in Equatorial Guinea, using blood samples and mosquitoes. The results showed that *P. vivax* seems to be able to invade erythrocytes using receptors other than Duffy, and this new capacity is not exclusive to one strain of *P. vivax*, since we have found samples infected with two different strains: VK247 and classic. Additionally we demonstrated that the parasite has a greater distribution than previously thought, calling for a reevaluation of its worldwide distribution.

## Introduction

*Plasmodium vivax* has been neglected by the scientific community since it has been seen as a “benign” parasite. Nowadays this scenario has changed and the infection caused by *P. vivax* gained higher importance, firstly because it has a very wide distribution, being found both in tropical and subtropical areas [1], [2], [3]; and secondly because of the high number of clinical cases reported, ranging from 70 million to 300 million [2], [4], [5]. Although clinical symptoms are usually considered as not severe, some reports documented cases of severe disease and even death [6], [7], [8], [9].

This parasite has traditionally shown a small prevalence in West and Central Africa, attributed to the high prevalence of Duffy negative people [Fy(a−b−)] who are described as being resistant to *P. vivax* infection [10], [11]. Culleton et al. [12] performed a study including nine endemic countries of West and Central Africa using a high sensitive PCR-based protocol for the detection and identification of *Plasmodium* species reporting only one case out of 2588 individuals infected with *P. vivax* - one Duffy-positive individual from São Tomé. Although the exact prevalence of *P. vivax* in Africa is unknown, this parasite tends to be endemic in countries of East Africa, like Sudan, Somalia and Ethiopia, where the majority of the population is Duffy-positive.

The Duffy antigen, also called Duffy antigen receptor for chemokines (DARC), is a multimeric red cell membrane protein organized into seven transmembrane domains, and it is the unique known erythrocyte receptor for *P. vivax* invasion. DARC-coding gene is polymorphic with multiple alleles as the codominant FY*A and FY*B, which encode for the two antigens – Fya and Fyb. Four genotypes are possible as a result of the combination of the major alleles, Fy(a+b+), Fy(a+b−), Fy(a−b+) and Fy(a−b−) [13], [14], [15]. The first three correspond to a Duffy-positive phenotype, mostly prevalent in Asian and in Caucasian populations and the last one correspond to the Duffy-negative phenotype, mainly prevalent in African people, who are consequently resistant to *P. vivax* infection. The Fy(a−b−) genotype results from a point mutation, -33T>C, in the promoter region of allele FY*B, in the GATA box region [13].

Recent data showed that Duffy binding protein, the main vaccine candidate for *P. vivax* [16], [17], seems no longer to protect against *P. vivax* infection. Rosenberg [18] hypothesized that *P. vivax* could infect Duffy negative erythrocytes, since there were reports of European travellers and immigrants from West and Central Africa who were infected with *P. vivax* [19], [20], [21]. In fact, there are now other reports that seem to support this hypothesis [18].

In a case-control study conducted in Kenya, an East African country, with children with severe malaria caused by *Plasmodium falciparum*, it was found that there were children infected with *P. vivax* VK247 despite being Duffy-negative [22]. Similar results were found in the Amazon region in Brazil [23], [24] and more recently in Madagascar [25]. These new data suggest that *P. vivax* may be evolving by using alternative receptors to bind and invade erythrocytes or it may be a “*vivax*-like” that do not require Duffy antigen for the invasion [26].

Currently, three different strains of *P. vivax* have been described – classic *P. vivax* (also called *P. vivax* VK 210), *P. vivax* VK 247 and *P. vivax-like*. These strains, although morphologically similar, differ in the central portion of circunsporozoite surface protein (*csp*), an abundant polypeptide present at the sporozoite surface [27]. The variant VK247 was first described by Rosenberg et al. [28] in isolates from Thailand and differs from the *P. vivax* classic in the nonapeptide repeat units of the central portion of CSP gene: ANGA(G/D)(N/D)QPG in *P. vivax* VK247 and GDRA(A/D)GQPA in *P. vivax* classic (described in [29]). Qari et al. [26] identified the strain *P. vivax-like*, characterised by having a 11-mer repeat sequence, APGNQ(E/G)GGAA in the central portion of the CSP gene.

With new cases of *P. vivax* infections appearing every day, especially in countries where this parasite has not been reported before, it becomes essential not to underestimate it, since *P. vivax* may be swiftly evolving and infecting people that were thought to be protected.

The aims of this study were to investigate the presence of *P. vivax* in Angola and in Equatorial Guinea, using blood samples and mosquitoes, and analyze the presence of *P. vivax* infection in Duffy-negative individuals.

## Methods

### Ethics statement

Each person (or parent) was informed of the nature and aims of the study and told that participation was voluntary and that they could withdraw from the study at any time. Blood samples were collected after informed consent from all donors (parents or guardians respond on behalf of children). In Equatorial Guinea, written consent was not obtainable because of the community-wide mistrust of signing any official forms and the low level of literacy in the population. Viewing this, written consent was only assented by population in case of the legal guardians of the recruited children and only non-documented oral consent was requested on adults. The study was approved by the Ethical Committee of the Equatorial Guinea's Ministry of Health and Social Welfare, the National Malaria Control Programme and the local health authorities from these villages, which accepted this constraint and did not find bio-ethical impediments to disallow the study. In Angola, written informed consent was obtained from each person (or parent/guardian) and the study was approved by the Ethical Committee of the Angola's Ministry of Health. Ethical clearance was also given by the Ethical Committees of IHMT and the ISCIII, according to EU norms.

### Sampling

**Figure 1:**
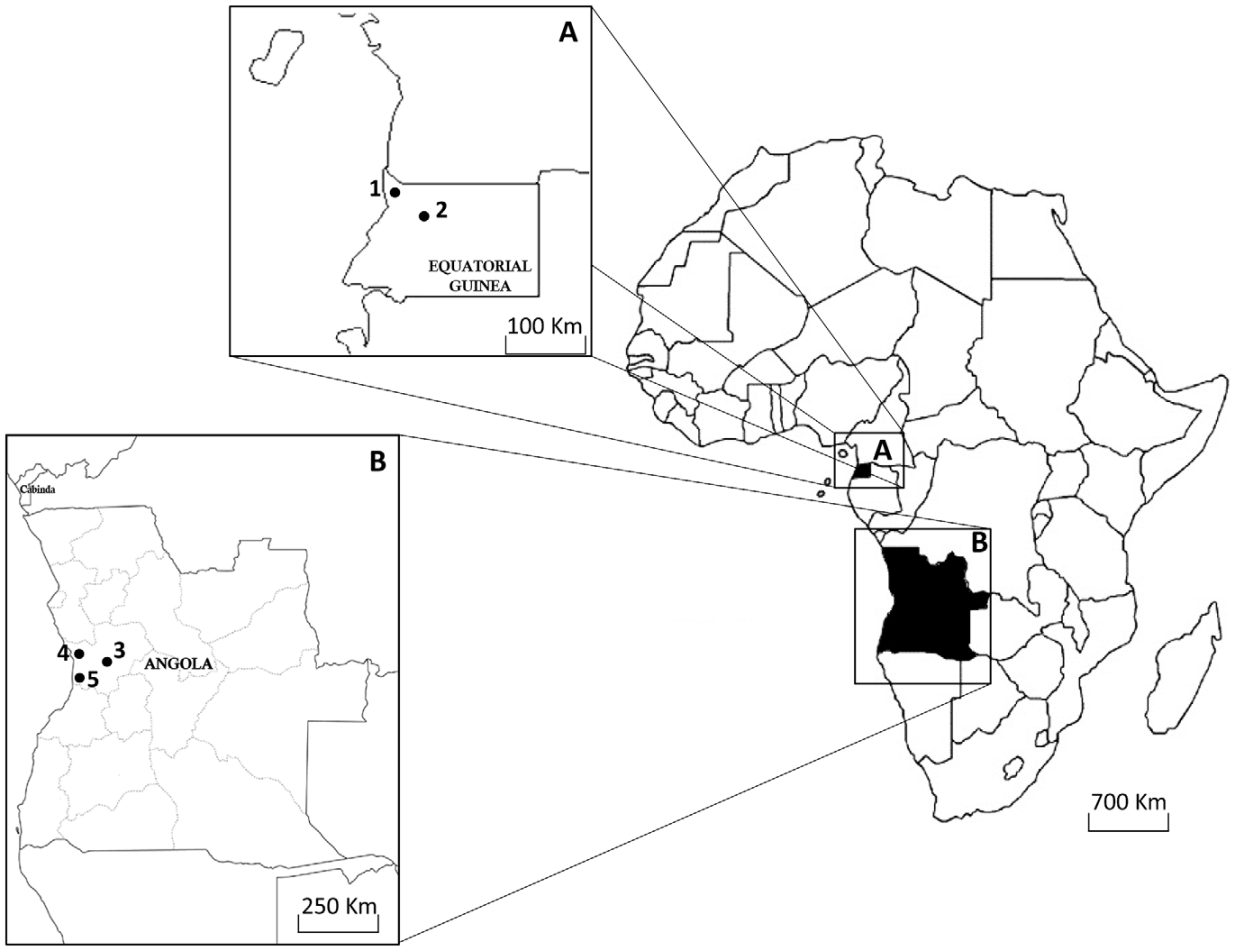
Map of the five collection places in Equatorial Guinea and Angola. A- Equatorial Guinea; B- Angola; 1- Ngonamanga; 2- Miyobo; 3- Gabela; 4 – Porto Amboim and 5- Sumbe.

Blood samples were collected as part of two on-going studies in Angola and Equatorial Guinea (see figure 1).

Angola samples were collected in Gabela (10°S51′/14°E22′), Porto Amboim (10°S43′/13°E46′), Kissala-Sumbe (11°S12′/13°E50′), Praia-Sumbe (11°S12′/13°E50′) [Kuanza Sul province] and Funda (8°S50′/13°E33′) [Bengo province] between June 2006 and May 2007. In these two provinces malaria is mesoendemic stable and the climate is tropical, characterised by a wet and warm season, from September to April, and a dry and cold season, from May to August. In each village, blood samples were collected by fingerprick on filter paper, from asymptomatic children older than 2 months.

In Equatorial Guinea, blood samples and mosquito specimens were collected from 20 households in two different villages - Miyobo (1°N45′/10°E10′) in May and August of 2005 and Ngonamanga (2°N9′/9°E48′) in February and May of 2005. The two villages present different ecological characteristics: Miyobo is located in the interior of the country in a forested area, while Ngonamanga is a coastal area. In both, malaria is classified as hyperendemic, and it is possible to distinguish four seasons, two dry seasons from December to March and from July to September; and two wet seasons, one more intense from September to November and the other from March to the end of June. In each household, blood fed resting mosquitoes were collected early in the morning (5.00–7.00am), followed by blood sample collection by fingerprick from all inhabitants, during four consecutive days. Mosquitoes were kept in paper-cups corresponding to each house/room for 8 days to enable the development of oocysts from infections acquired the night prior to collection. Head/thorax and abdomen from each mosquito were kept separately for subsequent molecular processing.

### Sample collection and DNA extraction

Blood samples from a total of 995 individuals (898 from Angola and 97 from Equatorial Guinea) were collected by fingerprick on filter paper and stored at room temperature until DNA extraction, which was carried out using the chelex protocol as described by Plowe et al. [30].

DNA from the 819 mosquitoes captured in Equatorial Guinea was extracted using the chelex protocol described by Arez et al. [31]. DNA from portions head/thorax and abdomen of each mosquito was extracted separately.

### Detection and identification of *Plasmodium* species

For all samples, detection of malaria infection and identification of *Plasmodium* species was made using nested-PCR amplification of the small subunit ribosomal RNA genes as described by Snounou et al. [32].

### Genotyping of Pvcsp genes

In samples infected with *P. vivax*, parasite characterisation was carried out by analysis of the central region of the *Pvcsp* gene, following a slightly modified version of the protocol described by Alves et al. [33]. This was amplified in a MyCyclerTM Thermal cycler (Biorad), using the primers VivF 5′- TCCATCCTGTTGGTGGACTT – 3′ and VivR 5′ – TCACAACGTTAAATATGCCAG – 3′ with final reagent concentrations of 1× reaction buffer, 1 mM of MgCl_2_, 100 µM of each dNTPs, 0.5 µM of each primer and 1 U/µl of Taq DNA Polymerase (Promega), in a total volume of 50 µl for each reaction. The PCR cycle conditions were: initial denaturation at 94°C for 5 minutes, followed by 35 cycles of 93°C for 1 minute, 60°C for 90 seconds and 72°C for 1 minute, with a final extension at 72°C for 10 minutes.

In order to distinguish the three *P. vivax* strains (VK210, VK247 and *P. vivax-like*), restriction fragment length polymorphism (RFLP) analysis was performed using the restriction endonucleases (AluI and DpnI), following the recommended protocol (New England Biolabs, Ipswich, MA). PCR-RFLP products were run in a 2% agarose gel.

### Genotyping of Duffy blood group

Duffy genotypes were also determined in *P. vivax* human isolates. To detect the point mutation -33T>C, which correspond to a Duffy-negative phenotype, the DARC gene promoter regions were amplified by PCR, followed by enzymatic restriction with StyI (New England Biolabs, Ipswich, MA) (adapted from [13]). Briefly, the PCR was performed using the primer P38 5′- AGGCTTGTGCAGGCAGTG - 3′ and P39 5′- GGCATAGGGATAAGGGACT - 3′, 0.5 pmol/µl of each, 1 mM of MgCl_2_, 200 µM of dNTP's and 1 U/µl of Taq DNA Polymerase (Promega), in a total volume of 30 µl. Cycling parameters were as follows: 94°C for 5 minutes, pursued by 30 cycles of 94°C for 1 minute, 59°C for 1 minute and 72°C for 30 seconds, with a final extension at 72°C for 10 minutes.

Endonuclease StyI was used for RFLP analysis of PCR products, according to the supplier's specifications (New England Biolabs, Ipswich, MA). Restriction fragments were separated on an 18% acrylamide/bis-acrylamide (39.5∶1) gel and silver stained.

For confirmation, some samples were purified with the SureClean Kit (Bioline) according to manufacturer's recommendations and were sequenced in both directions by Macrogen, Korea.

## Results

### Detection and identification of *Plasmodium* species

The four species of *Plasmodium* were identified in both countries. *Plasmodium vivax* had not been previously described in the mainland of Equatorial Guinea.

Prevalence of infection in both blood samples and mosquitoes is presented in Table 1. Regarding the human host, overall prevalence of infection was much higher in Equatorial Guinea than in Angola (86.6% *versus* 28.9%, respectively), with *P. falciparum* showing the highest infection rate in both countries (95.2% in Equatorial Guinea and 97.9% in Angola). *Plasmodium vivax* was detected in 15 individuals, 8 from Equatorial Guinea (9.5% of infected individuals) and 7 from Angola (2.8% of infected individuals). From these 15 cases, 5 exhibited a single *P. vixax* infection, 8 a mixed infection with *P. falciparum* and 2 a triple infection with *P. falciparum* and *Plasmodium malariae*. In Equatorial Guinea, the overall prevalence of infected mosquitoes was 26.7% (219/819). From these, *P. vivax* infections were found in 10.9% (24/219), both in head/thorax (salivary glands) and abdomen (midgut): 22 were a single *P. vixax* infection and 2 a mixed infection with *P. falciparum*.

**Table 1:** Prevalence of infection in both humans and mosquitoes, in Angola and Equatorial Guinea.

Prevalence of infection	Individuals		Mosquitoes
	Angola	Equatorial Guinea	
**n**	898	97	819
**Overall infection**	28.9% (245/848)	86.6% (84/97)	26.7% (219/819)
**Overall infection F**	97.9% (240/245)	95.2% (80/84)	89.0% (195/219)
**Overall infection V**	2.8% (7*/245)	9.5% (8*/84)	10.9% (24/219)
**V**	3 ind.	2 ind.	22 mosq.
**F+V**	3 ind.	5 ind.	2 mosq.
**F+V+M**	1 ind.	1 ind.	0

*All Duffy-negatives.

n - Sample size; F: *P. falciparum*; V: *P. vivax*; F+V: mixed infection by *P. falciparum* and *P. vivax*; F+V+M: mixed infection by *P. falciparum*, *P. vivax* and *P. malariae*.

### Genotyping of Pvcsp genes

Using the endonuclease AluI the fragments obtained for the *P. vivax* classic were: 243, 135, 133, 108, 90, 78, 57, 54, 30, 27 bp and for *P. vivax* VK247 were: 673, 243, 90, 78 bp. Using the endonuclease DpnI it was possible to identify fragments of 969, 71 and 50 bp in the case of *P. vivax* classic, and fragments of 360, 225, 108, 81, 71, 54, 50, 27 bp for *P. vivax* VK247. Fragments below 50 bp were not considered for variant determination due to the low molecular weight.

According to this, it was possible to identify 6 blood samples infected with *P. vivax* classic, 6 blood samples with *P. vivax* VK247 and 3 blood samples infected with two strains of *P. vivax*: classic and VK247.

No samples were identified as being infected with *P. vivax-like*. In this case, it was expected to obtain fragments of 786, 101, 83, 70 and 62 bp when using AluI, and fragments of 883, 169 and 50 bp when using DpnI.

For the 24 mosquitoes infected with *P. vivax*, the same procedure was used for the parasite characterisation but unfortunately no successful amplification of specific sequences was achieved.

### Genotyping of Duffy blood group

All the human isolates *P. vivax* infected were genotyped for the Duffy gene by PCR-RFLP (82, 77 and 64 bp for Duffy positive genotypes and 82, 65, 64 and 12 bp for Duffy negative genotypes; the fragment of 12 bp was not considered due to the low molecular weight, not visible in gel). Results showed that all samples analysed were genotyped as FY*B-33/FY*B-33 (Duffy-negative homozygous) being therefore classified as Fy(a−b−).

**Figure 2:**
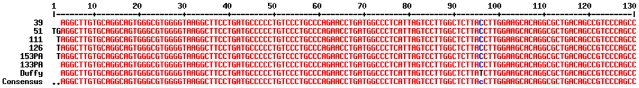
Multiple sequences alignment of promoter region from the DARC gene, allele FY*B, in the GATA box region.

Given that differentiation of bands in acrylamide gel is sometimes dubious, some samples were sequenced to confirm results. Sequencing (figure 2) confirmed the Duffy-negative genotype, since all of them contained the point mutation -33T→C.

## Discussion

Despite all the efforts that have been made to control malaria, many of them having a real effect, the prevalence of infection is still very high, even in countries with active control campaigns, like Equatorial Guinea (86.6%) and Angola (28.9%).

Particularly, *P. vivax* seems not only to be evolving and adapting, causing more severe forms of the disease [6], [8], [34], [35], [36] but also appears to be more frequent in countries where either it was not present or it was not detected by the available techniques in the past, as is the case of some countries of West and Central Africa like, Congo [37], São Tomé and Principe [37], [38], Gabon [37], [39] and Cameroon [37], becoming a major source of concern. Our results corroborate these assumptions, since for the first time we were able to detect *P. vivax* on mainland Equatorial Guinea in humans and mosquitoes, which imply well-established whole life-cycles and active transmission.

Further, a relevant aspect needs to be stressed - the proportion of *P. vivax* infected mosquitoes is higher than the proportion of *P. vivax* infected individuals. This may be associated with the fact that in the human host this parasite may be “hidden” since it forms dormant forms in the liver – hypnozoites - and go unnoticed, being much more “visible” in mosquitoes. If this is the case, these results suggest that the prevalence of this species may be underestimated, not only in this country but in other parts of Africa.

Other factors associated with parasite-human interaction and immune response could be conditioning this variable prevalence in *P. vivax* infection in mosquitoes and human host.

In this study we were able to detect Duffy negative people carrying *P. vivax* infections, both in Angola and Equatorial Guinea, two countries located in West Africa, where the prevalence of Duffy negative individuals is near 95% [11], confirming thereby the suspicion of some authors [18], [22]–[25]. Similar results were found in other studies, but always in areas where the prevalence of Duffy positive is significantly higher: in Kenya - East Africa [22], Amazon region in Brazil [23], [24] and more recently, in Madagascar [25].

Ménard *et al.* [25] suggested that Duffy positive individuals may serve as a reservoir of *P. vivax* providing an opportunity for this parasite to infect hepatocytes of Duffy negative people and the selection of new *P. vivax* strains with capacity to invade Duffy negative erythrocytes. In the present case, it is likely that the evolutionary process has been the same, although these two countries showed low prevalence of Duffy positive autochthonous individuals. From the beginning of the 90 s, these countries have experienced a marked increase in economic development with the finding of important oil reserves. Related to this development, intensive migration processes are occurring from outside and inside of the African continent. Therefore, workers from countries with higher Duffy positive and *P. vivax* prevalence could be circulating in Angola and Equatorial Guinea, thus increasing the reservoir of *P. vivax*.

Although we do not know which main force was contributing for the evolution of *P. vivax* and why it is able to infect Duffy negative erythrocytes, one thing seems to be clear - *P. vivax* may have an extraordinary ability to adapt. In addition, the African continent has both the ideal temperature and highly competent vectors for its transmission [3], [40]. Altogether, these factors show that this parasite can become a serious public health problem in West and Central Africa, both for locals and travelers.

The results obtained in this work are highly relevant. First, it demonstrates that *P. vivax* is able to invade erythrocytes using other receptors than Duffy, and this new capacity is not exclusive of one strain of *P. vivax*, since we found samples infected with two different strains: VK247 and VK210/classic. Other species of *Plasmodium*, as *Plasmodium knowlesi* (phylogenetically close to *P. vivax*) and *P. falciparum* have more than one receptor for the invasion of erythrocytes [41]. Considering that these two phylogenetically distant species have evolved in order to recognize more than one receptor for erythrocyte invasion, it is expected that *P. vivax* is also evolving, becoming capable of using more than one path of invasion.

Second, this parasite seems to be expanding, and now it can be found in areas where it was not present in the past. Some approaches to determine the distribution limits of *P. vivax* have been carried out, although areas with high prevalence of Duffy negative were virtually considered free of this parasite [3]. So it is expectable that the real distribution of this parasite is greater than that found by these authors.

In conclusion, this study present the first cases of Duffy negative individuals infected with different strains of *P. vivax* (VK247 and classic) in two West African countries. This finding reinforces the idea that this parasite is rapidly evolving, being able to use other receptors than Duffy to invade the erythrocytes.

The presence of *P. vivax* infection both in blood samples and mosquitoes indicates that this parasite is well adapted. Further, the higher number of infected mosquitoes shows that this species is more “visible” in mosquitoes and may go unnoticed if blood samples are only analyzed.

It is therefore important to establish the real distribution of *P. vivax*, since new and more aggressive cases of infection by this parasite are reported every day, in countries where this parasite has not been noticed before having significant implications in the design of control measures and implementation of prophylactic and therapeutic regimens.

## Supporting Information

Checklist S1 STROBE checklist.
(DOC)
